# Particulate air pollutants, APOE alleles and their contributions to cognitive impairment in older women and to amyloidogenesis in experimental models

**DOI:** 10.1038/tp.2016.280

**Published:** 2017-01-31

**Authors:** M Cacciottolo, X Wang, I Driscoll, N Woodward, A Saffari, J Reyes, M L Serre, W Vizuete, C Sioutas, T E Morgan, M Gatz, H C Chui, S A Shumaker, S M Resnick, M A Espeland, C E Finch, J C Chen

**Affiliations:** 1 Leonard Davis School of Gerontology, University of Southern California, Los Angeles, CA, USA; 2 Department of Preventive Medicine, Keck School of Medicine, University of Southern California, Los Angeles, CA, USA; 3 Department of Psychology, University of Wisconsin, Milwaukee, WI, USA; 4 USC Viterbi School of Engineering, University of Southern California, Los Angeles, CA, USA; 5 Department of Environmental Sciences and Engineering, Gillings School of Global Public Health, University of North Carolina at Chapel Hill, Chapel Hill, NC, USA; 6 Department of Psychology, University of Southern California, Los Angeles, CA, USA; 7 Memory and Aging Center, Keck School of Medicine, University of Southern California, Los Angeles, CA, USA; 8 Department of Neurology, Keck School of Medicine, University of Southern California,, Los Angeles, CA, USA; 9 Department of Social Sciences and Health Policy, Wake Forest School of Medicine, Winston-Salem, NC, USA; 10 Laboratory of Behavioral Neuroscience, National Institute on Aging, National Institutes of Health, Baltimore, MD, USA; 11 Division of Public Health Services, Department of Biostatistical Sciences, Wake Forest School of Medicine, Winston-Salem, NC, USA

## Abstract

Exposure to particulate matter (PM) in the ambient air and its interactions with APOE alleles may contribute to the acceleration of brain aging and the pathogenesis of Alzheimer's disease (AD). Neurodegenerative effects of particulate air pollutants were examined in a US-wide cohort of older women from the Women's Health Initiative Memory Study (WHIMS) and in experimental mouse models. Residing in places with fine PM exceeding EPA standards increased the risks for global cognitive decline and all-cause dementia respectively by 81 and 92%, with stronger adverse effects in APOE ɛ4/4 carriers. Female EFAD transgenic mice (*5xFAD*^*+/−*^*/human APOE* ɛ*3* or ɛ*4*^*+/+*^) with 225 h exposure to urban nanosized PM (nPM) over 15 weeks showed increased cerebral β-amyloid by thioflavin S for fibrillary amyloid and by immunocytochemistry for Aβ deposits, both exacerbated by *APOE* ɛ4. Moreover, nPM exposure increased Aβ oligomers, caused selective atrophy of hippocampal CA1 neurites, and decreased the glutamate GluR1 subunit. Wildtype C57BL/6 female mice also showed nPM-induced CA1 atrophy and GluR1 decrease. *In vitro* nPM exposure of neuroblastoma cells (N2a-APP/swe) increased the pro-amyloidogenic processing of the amyloid precursor protein (APP). We suggest that airborne PM exposure promotes pathological brain aging in older women, with potentially a greater impact in ɛ4 carriers. The underlying mechanisms may involve increased cerebral Aβ production and selective changes in hippocampal CA1 neurons and glutamate receptor subunits.

## Introduction

Environmental influences on Alzheimer's disease (AD) and related dementias (ADRD) are poorly documented.^1^ Apolipoprotein E (*APOE*) ɛ4 and other loci identified by large GWAS account for less than 50% of heritable AD risk.^2^ Thus, attention is drawn to environmental risk factors, including common neurotoxins and their interactions with APOE and other genes.^2, 3^

Ambient fine particles (PM_2.5_: particulate matter (PM) with aerodynamic diameters<2.5 μm) from traffic emissions are a major source of urban air pollution, accounting globally for 25% of ambient PM_2.5_.^4^ Epidemiologic evidence associates cognitive deficits with PM_2.5_ exposure among the elderly.^5^ Rodent models also show long-term neurotoxic effects of air pollutants, including memory impairment^6^ and selective atrophy of CA1 hippocampal neurons observed in pre-clinical AD;^7^ decreased glutamate receptor subunit GluR1;^8^ and increased endogenous soluble Aβ.^9, 10, 11^ However, we lack prospective studies of PM exposure on ADRD risk and interaction with *APOE* alleles.

We hypothesized that long-term PM_2.5_ exposure increases the risk for accelerated global cognitive decline and dementia, further exacerbated by APOE ɛ4. These hypotheses were tested within the Women's Health Initiative Memory Study (WHIMS), a well-characterized, nationwide prospective cohort of older US women, for which we recently reported associations between elevated PM_2.5_ and smaller white matter volumes in multiple brain regions.^12^ Neurotoxic effects of PM were studied with transgenic mice (EFAD) carrying human APOE alleles and familial AD genes^13, 14^ which model pre-clinical accumulations of Aβ amyloid and its exacerbation in APOE ɛ4 carriers.^13, 15, 16^ We focused on female mice, because ɛ4 confers a greater AD risk in women than in men^15^ and because women also incur worse cardiopulmonary^17^ and neurological^18^ consequences from residential exposure to ambient PM.^17^ To model the human subpopulation with low to negligible Aβ plaque and without familial AD genes, we examined C57BL/6J mice (wildtype), which do not develop amyloid aggregates at any age, because murine Aβ differs from the human in 3 residues that reduce its aggregation.^19^ Nonetheless, sAPPα, derived from the endogenous amyloid precursor protein (APP), modulates synaptic remodeling.^20, 21^ We also examined responses of mouse neuroblastoma N2a cells expressing Swedish mutant APP (N2a-APP/swe) to *in vitro* nPM as a model for direct effects of PM on APP processing.

## Materials and methods

### The neuroepidemiologic study

WHIMS participants were community-dwelling (>95% in urban areas) across 48 states, aged 65 to 79 years, and free of dementia when enrolled, 1995–1999. Of 4504 with APOE genotypes, we excluded 717 with ɛ2/2, ɛ2/3 or ɛ2/4 allele plus 140 with missing PM_2.5_ data. The remainder of 3647 older women with APOE alleles ɛ3/3 (*n*=2644), ɛ3/4 (*n*=922) or ɛ4/4 (*n*=81), were of European ancestry (primarily non-Hispanic whites) and had complete PM_2.5_ exposure estimates. The standardized WHIMS outcome ascertainment protocols^22^ consisted of annual screening of global cognitive function, neuropsychological and functional assessment, with clinical data to exclude possible reversible causes of cognitive impairment (Supplementary Material), all concluded with the final classification of dementia (vs non-demented) by central adjudication blind to estimated PM_2.5_ exposure. Accelerated decline in global cognitive function was defined by an 8-point (~2 s.e.) loss in Modified Mini-Mental State (3MS)^23^ during two consecutive assessments. Decrease of 3MS by 5–10 points was considered a clinically significant decline in global cognitive functions.^24^

Using the Bayesian Maximum Entropy method (Supplementary Information), we constructed spatiotemporal models to estimate the ambient concentration of PM_2.5_ at all WHIMS residential locations in 1999–2010.^25^ This method integrates nationwide monitoring data from the U.S. EPA Air Quality System (AQS) and the output of chemical transport models to characterize spatiotemporal interdependence of environmental data to estimate mean trends and covariance of the air pollution fields over space and time. The resulting BME estimates of daily PM_2.5_ exposures correlated with levels recorded at local AQS monitoring sites (cross-validation Pearson's *r*^2^=0.70). This statistically-validated BME model was applied to each geocoded residential location to generate a yearly time-series of PM_2.5_ exposure, and then combined with residential histories including relocations to calculate the 3-year moving average PM_2.5_ exposures.

### Statistical analysis

We conducted time-to-event analyses to examine associations between long-term residential exposure to PM_2.5_ and adverse neurocognitive outcomes. Cox proportional hazard models were used to estimate hazard ratios (HRs) and 95% confidence intervals (CIs) for adverse events associated with estimated time-varying 3-year average PM_2.5_ exposures, adjusting for potential confounders, including age, geographic region, education, income, employment status, lifestyle factors (smoking; alcohol use; physical activities) and clinical characteristics (use of hormone treatment; depression; body mass index; hypercholesterolemia; hypertension, diabetes; and histories of cardiovascular disease). Characterization of these covariates and rationale for their selection were given in the Supplementary Information. Follow-up time for each woman was calculated from WHI randomization (baseline) to the screening date triggering the ultimate classification of defined outcome end points, or the last date of completing annual cognitive assessment before 31 December 2010, whichever came first. Data on global cognitive decline and incidence of dementia were analyzed separately. For analyses of global cognitive decline, dementia cases were excluded if ascertained before subjects lost 8 points on 3MS. We used time on study as the timescale in the constructed Cox regression models, because simulation studies suggested that such approaches were less subject to potential biases in estimating effects of environmental factors (for example, PM exposures) with prominent secular trends,^26^ as compared with the other alternatives (for example, attained age; calendar time). The assumed proportional hazards of Cox models were supported by the proportionality test based on weighted residuals.^27^ To evaluate the effect measure modification, we further stratified the effect estimates by examining whether the putative neurotoxic effects differed by APOE alleles, by Wald tests of interaction. Statistical analyses used SAS System for Windows, Version 9.3 (SAS Institute, Cary, NC, USA).

The institutional review boards of all institutes involved in the air pollution neuroepidemiologic study and its parent projects approved the established protocols of human subject protection and informed consent.

### Mouse experiments

#### Animals

EFAD mice carrying transgenes for human APOE ɛ3 or ɛ4 alleles in combination with five familial AD mutations (*5xFAD*^*+/−*^*/human APOE*^*+/+*^) (APP K670N/M671L+ I716V+ V717I and PS1 M146L+L286V)^13^ were generously given by Dr Mary Jo LaDu (University of Illinois, Chicago, IL, USA). Experimental logistics limited the exposure study to 20 female mice: 10 per group of E3- and E4FAD, were randomly assigned to either nPM exposure or control air for 15 weeks. One E3FAD control died during the experiment. As a model for the human subpopulation with low to negligible Aβ plaque and without AD genes, we exposed wild-type C57BL/6J females (*n*=18) to nPM for 10 weeks corresponding to our prior study of wild-type male mice.^8^ The 15-week exposure for EFAD was chosen to initiate exposure at 2 months, corresponding to the onset of Aβ deposition,^13^ with brains collected at age of 7 months, the same age in our initial study.^8^ Data analysis was blinded for nPM and genotype. Mouse husbandry and procedures were approved by the University of Southern California Institutional Animal Care and Use Committee.

#### Experimental exposures

A nano-scale subfraction of urban PM_2.5_, designated as nPM^8^ with well-characterized particle size and chemical composition,^8, 28^ was used for *in vivo* and *in vitro* exposure. Female mice were randomly assigned to nPM or filtered air (control), 5 h per day, 3 days per week, delivered to the sealed exposure chambers. For timelines of exposures see Supplementary Figure 1.

#### Tissue collection

Mice were killed by isoflurane anesthesia and perfused transcardially with phosphate-buffered saline. Brains were hemi-sected for sagittal sectioning 0.5–2 mm from midline. Brains were fixed in 4% paraformaldehyde, cryoprotected in sucrose and frozen on dry ice. The other hemisphere was chilled and dissected (hippocampus and cerebral cortex) and frozen on dry ice.

#### Oligomeric Aβ ELISA

Aβ peptides were assayed in brain supernates.^15^ Cerebral half-cortexes were homogenized in DEA buffer (0.2% diethylamine, 50 mM NaCl; 1 ml per 200 mg tissue) with Complete Protease Inhibitor (Sigma, St. Louis, MO, USA). After centrifugation (20 800 *g* × 30 min), supernatants were neutralized with Tris-HCl, pH 6.2. Oligomeric Aβ was assayed by MOAB-2 ELISA kit (BEK-2215-1P, Biosensis, Thebarton, SA, Australia).

#### Aβ Immunohistochemistry (4G8)

Aβ amyloid was immunostained with 4G8 antibody (residues 17–24 at N-terminal of APP, SIG-39220, Covance, Princeton, NJ, USA).^15^ Briefly, sections were immersed in 70% formic acid/5 min. Endogenous peroxidases were blocked by 3% H_2_O_2_ and 10% methanol in TRIS-buffered saline (TBS), 30 min/22 °C. Sections were permeabilized in 0.1% Triton X-100/15 min, blocked by 30 min incubation in TBS with 2% BSA and 0.1% Triton, and probed with primary antibodies. After 0.1% Triton and TBS rinses, sections were incubated with biotinylated anti-mouse secondary antibodies (1:250) for 1 h, followed by ABC peroxidase and 3,3'-diaminobenzidine (DAB; Vector, Burlingame, CA, USA). Bright-field microscope images were converted to 8-bit grayscale and thresholded to highlight plaques and to diminish background. The objects identified were inspected individually to confirm plaque identity. The cerebral cortex in each image was outlined for analysis by ‘analyze particles' function in NIH ImageJ software. Aβ plaque load was evaluated as % area covered by 4G8-stained plaques.

#### Thioflavin S staining

Sections were air-dried, rehydrated in Milli-Qwater for 2 min and stained in 0.1% thioflavin S (ThioS) (in 50% ethanol-phosphate-buffered saline) for 5 min in the dark. Sections were destained twice for 5 min in 80% EtOH in the dark and mounted with Fluoromount Aqueous (Sigma Aldrich, St. Louis, MO, USA). Amyloid load was quantified as above for 4G8 immunostaining.

#### Silver staining

Silver staining Bielschowsky technique was used to assess neuropil density.^29^ Sections were dried at room temperature and briefly washed in distilled water before preimpregnation. Sections were incubated in preheated 20% (1.0 m) silver nitrate at 37 °C/15 min, washed 3 × in distilled water, and incubated in ammoniacal silver solution (20% silver nitrate in 148 mm ammonia water) for 10 min at 37 °C. Sections were developed for 3 min (8% formaldehyde, 1% nitric acid, 26 mm citric acid, diluted 1:50 in ammonia water), followed by washing in ammonia water and distilled water to reduce background. Slides were then placed in 5% sodium thiosulfate solution for 2 min, rinsed 5 × in distilled water, dehydrated, cleared and mounted. Bright-field images of CA1, CA2/3 and dentate gyrus region, were analyzed by NIH ImageJ software. Images were converted to 8-bit grayscale, thresholded for binary separation of neuronal cell bodies (dark round objects) from neurites and neurite density calculated as percentage of positive staining in area of interest.

#### Immunoblotting

Hippocampus was homogenized by motor-driven pestle in cold RIPA buffer (20–188, Millipore, Temecula, CA, USA) and centrifuged 5 min/20 000 *g*. Supernate protein (20 μg) was electrophoresed on 10% SDS polyacrylamide gels and transferred to polyvinylidene fluoride membranes. The polyvinylidene fluoride membranes were blocked with 5% BSA for 1 h and probed with primary antibodies overnight at 4 °C: anti-GluR1 (glutamate receptor subunit 1; 1:3000, AB31232, rabbit; Abcam, Cambridge, MA, USA), anti-GluR2 (glutamate receptor subunit 2; 1:2000, AB1768, rabbit; Millipore), anti-NR2A (NMDA receptor, subunit 2A, 1:1000, 07-632, rabbit; Millipore), anti-NR2B (NMDA receptor, subunit 2B, 1:1000, 06-600, rabbit; Millipore) anti-PSD95 (1:1000, AB2723, mouse; Abcam), anti-synaptophysin (1:5000, MAB368, mouse; Stressgene; Enzo, Plymouth Meeting, PA, USA), and anti-NueN (loading control; 1:1000, MAB377, mouse, Millipore). After washing, membranes were probed with secondary antibodies conjugated with IRDye 680 (92632210, rabbit, LI-COR Biosciences, Lincoln, NE, USA) and IRDye 800 (92632210, mouse, LI-COR). Signal was detected by infrared imaging (Odyssey, LI-COR).

#### *In vitro* nPM exposure and APP/Aß measurements

Mouse neuroblastoma N2a cells expressing Swedish mutant APP (K595N/M596L) (N2a-APP/swe) were generously gifted by Dr Huaxi Xu (Sanford/Burnham Medical Research Institute, La Jolla, CA, USA) and tested for mycoplasma contamination before use. Cells were treated with nPM (10 μg ml^−1^) in culture media (Optimem/DMEM medium, 5% FBS, 500 μg ml^−1^ G418) for 24 h. RIPA buffer cell lysates were probed with 22C11 antibody (1:100, Millipore), which recognizes both sAPPα and sAPPβ. Media were analyzed for Aβ42 by MSD Multiplex ELISA (Meso Scale Discovery, Rockville, MD, USA). Three independent experiments were performed, with three sample replicates each.

#### Statistical analyses

For the statistical analyses examining the putative effects on continuous response variables, we used multiple linear regression analysis in STATA14, including both nPM exposure and APOE genotype. We also conducted subgroup data analyses on nPM exposure effect, stratified by genotype. Silver staining analysis used repeated measurements of a clustered linear regression. All two-sided tests of statistical significance were set at *P*<0.05.

## Results

### The neuroepidemiologic study

Women in the highest PM_2.5_ quartile (14.34–22.55 μg m^−3^) were older (aged ⩾75 years); more likely to reside in the South/Midwest and use hormonal treatment; but engage less in physical activities and consume less alcohol, relative to counterparts (all *P*-values<0.05; Supplementary Table S1). PM_2.5_ exposure estimates did not differ by APOE genotype. There were 173 subjects classified as incident cases of all-cause dementia over an average follow-up of 9.9 years and 329 had global cognitive decline (including 87 cases subsequently classified as or progressing to all-cause-dementia) over an average follow-up of 8.3 years. The observed incidence rates of accelerated global cognitive decline and all-cause dementia differed significantly by APOE genotype (both *P*<0.001 with ɛ4/4>ɛ3/4>ɛ3/3 by log-rank test).

#### Associations between PM_2.5_ exposure and adverse events

**Figure 1:**
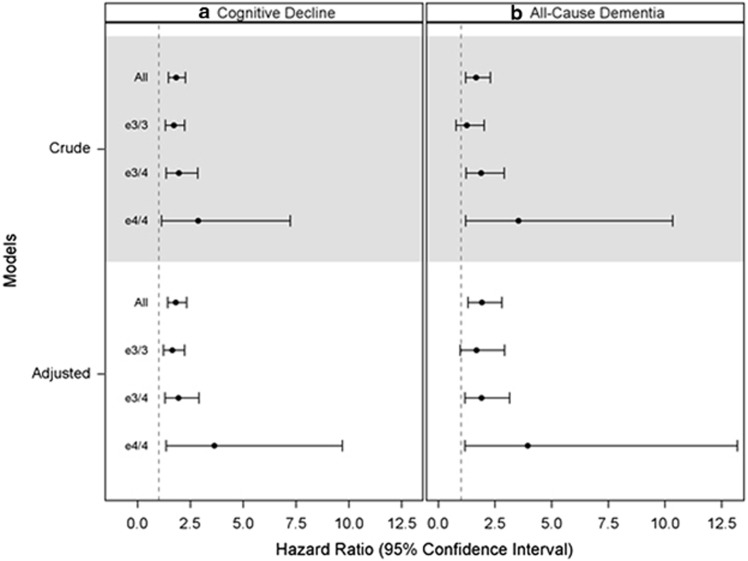
Adverse effects of PM_2.5_ exposure on cognitive impairment in older women, stratified by APOE alleles. Horizontal bars represent the effect measures (hazard ratios (HRs) and 95% confidence intervals) estimated from the Cox proportional hazard models, comparing high (exceeding the US National Ambient Air Quality Standard with 3-year averages PM_2.5_>12 μg m^−3^) versus low exposure for their associated incidence rates of global cognitive decline (**a**) and all-cause dementia (**b**), stratified by APOE alleles (ɛ3/3 vs ɛ3/4 vs ɛ4/4). The dotted vertical lines denote no statistically significant adverse effects (with HR=1). The presented crude estimates were adjusted for APOE alleles. The adjusted estimates further accounted for age, geographic region, spatial random effect, years of education, household income, employment status, lifestyle factors (smoking; alcohol use; physical activities) and clinical characteristics (use of hormone treatment; depression; body mass index; hypercholesterolemia; hypertension, diabetes; and histories of cardiovascular disease). At any time during 1999–2010, if older women were residing at locations with high PM_2.5_, their hazards for accelerated global cognitive decline and all-cause dementia respectively would be 81% (HR=1.81; 1.42–2.32) and 92% (HR=1.92; 1.32–2.80) greater than if they had low exposure. This increase in hazard for all-cause dementia associated with high PM_2.5_ exposure was 68% (HR=1.68; 0.97–2.92), 91% (HR=1.91; 1.17–3.14), and 295% (HR=3.95; 1.18–13.19), respectively, in ɛ3/3, ɛ3/4, and ɛ4/4 carriers. High PM_2.5_ exposure also increase the hazard for global cognitive decline by 65% (HR=1.65; 1.23–2.23), 93% (HR=1.93; 1.29–2.90), and 264% (HR=3.64; 1.36–9.69) in women of ɛ3/3, ɛ3/4, and ɛ4/4 alleles.

The 3-year average exposure preceding the incident event time was *a priori* classified as ‘high' if exceeding the current National Ambient Air Quality Standards (NAAQS) for PM_2.5_ (>12 μg m^−3^).^30^ Residence in high PM_2.5_ locations was associated with increased risks of global cognitive decline and all-cause dementia. These adverse PM_2.5_ effects were exacerbated among women of ɛ4/4 (Figure 1; Supplementary Table S2). For residence in locations with high PM_2.5_ at any time during 1999–2000, the hazard ratio (HR) for accelerated global cognitive decline and all-cause dementia were increased by 81% and 92%, respectively (Figure 1). These adverse PM_2.5_ effects varied by APOE allele, with ɛ3/3<ɛ3/4<ɛ4/4 for both global cognitive decline (ɛ3/3: HR=1.65; ɛ3/4: HR=1.93; ɛ4/4: HR=3.95) and all-cause dementia (ɛ3/3: HR=1.68; ɛ3/4: HR=1.91; ɛ4/4: HR=2.95).

### Mouse experimental studies

**Figure 2:**
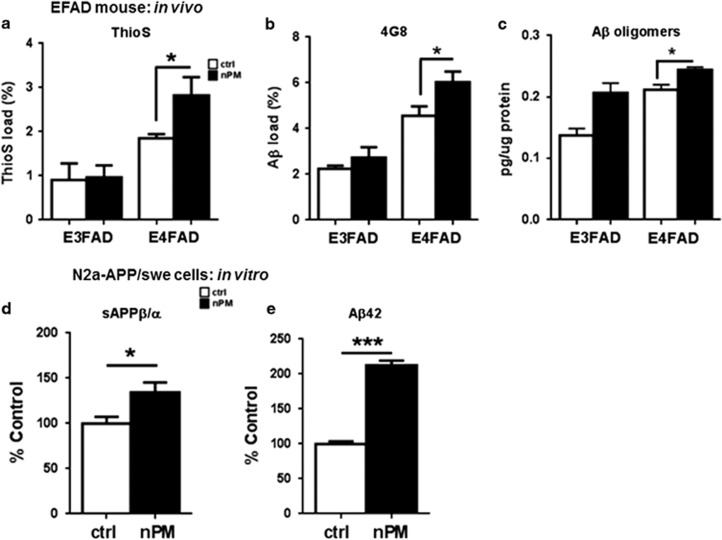
*In vivo* and *in vitro* nPM exposure on Aβ levels. (**a**,**b**) *In vivo* nPM exposure of female EFAD mice (*N*=5 mice per experimental group). (**a**–**c**). Cerebral cortex sagittal sections were analyzed for Aβ plaque load using two independent staining: (**a**) Thioflavin S, (**b**) 4G8 antibody. Both reagents showed responses to nPM in E4FAD mice but not in E3FAD. For Thioflavin S, E3FAD coeff, −0.09, *P*=0.79; E4FAD, coeff 1.06, *P*=0.048. E4FAD mice had 2.8-fold greater increased total plaque load after nPM than E3FAD mice (E3FAD coeff, 0.49, *P*=0.27; E4FAD, coeff 1.39, *P*=0.04). (**c**) Aβ oligomers in soluble extracts of cerebral cortex were increased by nPM exposure in E4FAD mice (coeff 0.03, *P*=0.03), with trend of increase in E3FAD (coeff 0.07, *P*=0.07). (**d**,**e**) *In vitro* nPM exposure (N2a-APP/swe cells). Cells exposed to 10 μg ml^−1^ nPM for 24 h showed 35% increased sAPPβ/α ratio (*P*=0.02). Culture media Aβ42 levels increased twofold (*P*<0.001). White bar, control; black bar, nPM exposed. Mean±s.e. *P<0.05, ***P<0.0001.

Female EFAD mice (*5xFAD*^*+/−*^*/human APOE* ɛ*3/*ɛ*3/* or ɛ4/ɛ*4*) were chronically exposed to nPM during 15 weeks. We observed increased amyloid deposits as fibrillar amyloid by ThioS binding and by 4G8 plaque immunohistochemistry that were greater for E4FAD mice than E3FAD. For ThioS, nPM exposure increased amyloid load by +60% in E4FAD above non-exposed controls (*P*=0.048), whereas E3FAD did not respond (*P*=0.79; Figure 2a). For 4G8, nPM exposure in E4FAD increased Aβ plaque load by +30% above controls (*P*=0.04; Figure 2b); its effect size was 2.8-fold greater than for E3FAD (Supplementary Table S3). Levels of ThioS and 4G8 were highly correlated (*r*^2^=0.78, *P*<0.0001; Supplementary Figure 2).

Aβ oligomers were increased by nPM for both APOE alleles, with an overall nPM effect (*P*=0.0001; Figure 2c): +15% in E4FAD mice (*P*=0.03) and +60% in E3FAD (*P*=0.07).

The increased amyloid from nPM exposure suggests direct effects of nPM on APP processing. As an acute *in vitro* model, mouse neuroblastoma cells (N2a-APP/Swe) were exposed to nPM; pro-amyloidogenic APP processing was assessed as the ratio (sAPPβ/α) of soluble fragments from β-secretase (sAPPβ, pro-amyloidogenic) and α-secretase (sAPPα, non-amyloidogenic). nPM increased the sAPPβ/α ratio by 35% (*P*=0.02, Figure 2d), with 2-fold increase of Aβ42 peptide (*P*<0.001, Figure 2e).

**Figure 3:**
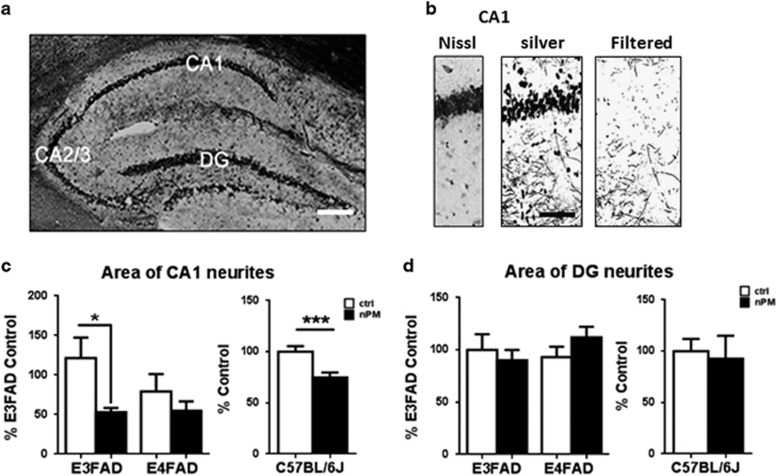
*In vivo* nanosized particulate matter (nPM) exposure decreased hippocampal CA1 neurite density. (**a**–**d**) Silver histochemistry for neurodegeneration in hippocampal subregions CA1 pyramidal neuron layer and dentate gyrus (DG). EFAD mice, *N*=5 mice per group; B6 mice, *N*=9 mice/group. (**a**) Whole hippocampus; scale bar, 500 μm. (**b**) Hippocampal subregions: scale bar, 100 μm; left panel: CA1, Nissl-stained CA1 neuron layer; center, detail of silver staining from Figure 3a to show neurites; right, density filtered to resolve neurites. (**c, d**) nPM caused decreased neurite density in CA1 neurons of EFAD and wild-type mice (C57BL/6J) without affecting dentate gyrus neurons. Overall nPM effects on CA1, combining E3FAD and E4FAD, was significant (*P*=0.02, adjusting for genetic effect): E3FAD (coeff=−0.09, *P*=0.03); C57BL/6J, (coeff=−0.25 *P*=0.003). White bar, control; black bar, nPM exposed. Mean±s.e. *P<0.05, ***P<0.005.

Neuronal consequences of the elevated Aβ and sAPPβ/α ratio include synaptic remodeling^20^ and effects of Aβ on glutamate receptors.^31^ The hippocampus of female EFAD and C57BL/6 mice showed selective neuronal atrophy in response to nPM, which was restricted to CA1 hippocampal neurons (Figure 3a and d): E3FAD (−45%, *P*=0.03) and C57BL/6 (−25%, *P*=0.003), without CA2/3 layer or dentate gyrus (Figure 3d, Supplementary Table S3).

**Figure 4:**
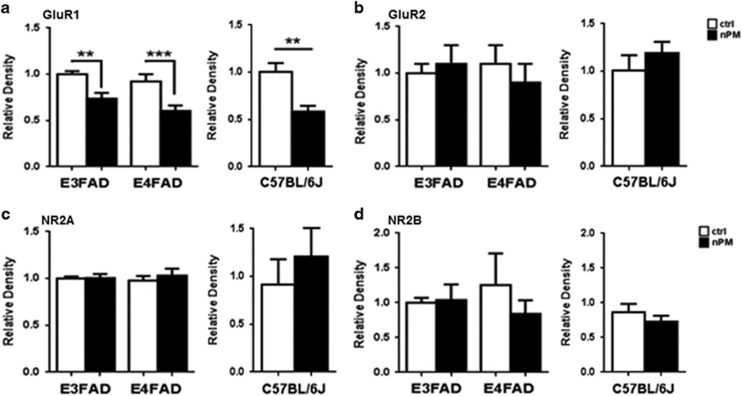
Chronic nPM exposure of female EFAD and C57BL/6J mice alters the GluR1 receptor subunit, but not other synaptic proteins. (**a**) Hippocampus glutamatergic receptor protein subunit GluR1 was decreased by nPM in both E3FAD (coeff=−0.26, *P*=0.01), E4FAD (coeff=−0.32, *P*=0.01) and C57BL/6J (coeff=−0.42, *P*=0.002) mice. (**b**–**d**) GluR2, NR2A and NR2B were unchanged. White bar, control; black bar, nPM exposed. EFAD, *N* = 5 mice per experimental group; B57BL/6, *N* = 9 mice per experimental group. Mean±s.e. **P<0.01, ***P<0.005.

Synaptic proteins that mediate hippocampal-based memory were analyzed in whole hippocampus extracts. The nPM exposure decreased GluR1 protein by −25% in E3FAD (*P*=0.01), −35% in E4FAD (*P*=0.01) and −40% in C57BL/6J (*P*<0.002; Figure 4a). No changes were detected in other glutamatergic receptor subunits (GluR2, NR2A and NR2B; Figure 4b–d) or other synaptic proteins (pre-synaptic: synaptophysin; post-synaptic: PSD95; Supplementary Figure 3).

## Discussion

Our data combine an air pollution-neuroepidemiologic study of older women and inhalation neurotoxicological experiments with mice. Together, we show the contribution of particulate air pollutants to neurodegenerative changes, with potentially a greater impact on APOE ɛ4 carriers. Overall, the evidence supports the schema that airborne PM accelerates neurodegenerative processes of ADRD through multiple pathways, including pro-amyloidogenic APP processing and other pathways independent of amyloid deposits.

In the geographically diverse WHIMS cohort, increased risks for both all-cause dementia and clinically significant declines in global cognitive function (with >8-point loss in 3MS scores) were associated with residential exposure to high levels of ambient PM_2.5_. In female EFAD mice, the chronic exposure to nPM, a neurotoxic subfraction of PM_2.5_, increased both the cerebral Aβ-amyloid plaque load and neurotoxic Aβ oligomers. Mice carrying ɛ4 had more nPM-induced Aβ-amyloid plaque. These experimental data are consistent with our epidemiologic observation of stronger associations between PM_2.5_ exposure and increased risks for dementia and global cognitive decline in women homozygous for APOE ɛ4 vs ɛ3. These findings provide the first experimental evidence for gene-environment interactions involving airborne PM and APOE in neurodegenerative processes.

Our study of the WHIMS cohort provides new evidence for late-life exposure to PM_2.5_ as a common environmental risk factor for ADRD. Previous studies showed older adults living in areas with higher ambient PM_2.5_ had lower performance in various cognitive functions^32, 33^ and accelerated cognitive aging.^34, 35^ However, unlike our defined global decline (>8-point loss in 3MS), the clinical significance was unclear for these reported cognitive deficits associated with air pollution exposure. Five studies that reported associations of ADRD with exposure to ambient air pollution^36, 37, 38, 39, 40^ had notable methodological limitations. Three of these studies^39, 40, 41^ used claims data to determine incident dementia/AD (an approach with questionable validity^42^) and included only aggregated exposures prone to ecological biases. Four studies^36, 39, 40, 41^ were retrospective and subject to selection biases.^43^ The only prospective cohort study^37^ employed a spatial model towards the end of study follow-up to estimate the NO_x_ exposure in earlier years, which obscured the temporality of the reported association. Our study within the prospective WHIMS cohort used the incident dementia cases adjudicated with well-validated protocols and a sophisticated spatiotemporal model to estimate residence-specific exposure to ambient PM_2.5_ preceding the ascertained end points. The comprehensive list of covariates data (including APOE genotype) allowed us to carefully assess and adjust for potential confounding. These observed neurodegenerative effects of PM_2.5_ (with the relative risk for global cognitive decline and all-cause dementia, respectively, increased by 81% and 92%) were not explained by differences in socioeconomic status, lifestyle, vascular risk factors and APOE alleles. The average PM_2.5_ concentrations have decreased over time in the US (for example, 34% reduction in 2000–2013), which coincided with decreased age-specific risk for dementia.^44^ Assuming 30% of older women in the US were residing in locations with high PM_2.5_ before the US EPA set its current US NAAQS standard ambient PM_2.5_ in 2012, if the observed adverse effects in WHIMS were generalizable, we estimate that ~21% of accelerated cognitive decline and all-cause dementia are attributable to residential exposure to high ambient PM_2.5_.^45^

The experimental findings with EFAD and C57BL/6J mice suggest possible mechanisms for the PM-associated cognitive impairment observed in the WHIMS cohort. We show that nPM exposure of EFAD mice increased fibrillary amyloid and Aβ plaque and soluble oligomeric Aβ, together with neuronal changes in hippocampus (discussed below). These are the first studies on the neurotoxicity of airborne particles using transgenic mice carrying human APOE alleles and familial AD genes. Three reports of wild-type rodents, including two with exposure to diesel particles^9, 10^ and one with concentrated ambient PM_2.5_,^11^ showed increased endogenous soluble Aβ in cerebral cortex. We also introduce an *in vitro* model for PM effects on APP processing. *In vitro* nPM short-term exposure of neuronal N2a-APP/Swe cells enhanced pro-amyloidogenic APP processing, with increased sAPPβ/α ratio and Aβ42 production. This finding concurs with the rapid rise of brain Aβ40–42 in wild-type mice exposed to nickel-PM enriched ambient air.^46^ In EFAD mice, Aβ plaques begin to form in cerebral cortex by 2 months,^13^ which models the pre-clinical accumulation of Aβ plaque in humans. We showed that nPM exposure increased both plaque formation and neurotoxic Aβ oligomers in cerebral cortex at 7 months (approximately equivalent to 35 years of human age, approaching peri-menopause). This implies that Aβ-dependent neurodegenerative processes in women with increased long-term PM exposure may precede cognitive declines or diagnosis of dementia, which is consistent with neuroimaging evidence for Aβ deposition even among cognitively intact individuals in their 50–60s.^47^

Importantly, nPM-exposed EFAD mice showed selective neuritic changes that parallel human AD, with selective atrophy of hippocampal CA1 neurons, but not of neighboring neurons of the same memory circuit.^7^ The nPM exposure also decreased the GluR1 subunit of glutamatergic synapses for both APOE alleles. Female wild-type C57BL/6 mice also showed selective CA1 atrophy with decreased GluR1, extending our prior findings of GluR1 with male C57BL/6J mice.^8^ Together, these results suggest that the nPM-associated early neurodegenerative changes in hippocampus may occur to a broader population regardless of underlying genetic risks.

The CA1 neurite atrophy in female C57BL/6J mice after 10 weeks of nPM exposure corroborates dendritic spine loss in CA1 neurons of male C57BL/6 mice exposed to concentrated ambient PM_2.5_ for 10 months shown by others.^6^ Thus, the selective vulnerability of CA1 neurons to airborne PM does not depend on the presence of human Aβ. As noted in the Introduction, wild-type murine sAPPα, derived from the endogenous mouse APP, modulates synaptic remodeling.^20, 21^ The selective CA1 attrition by PM exposure without Aβ accumulation implies that PM exposure before older ages could contribute to accelerated cognitive decline and increased AD risk in late life by reducing the CA1 synapses, possibly via direct interactions of Aβ oligomers with glutamatergic neurons.^31^ Moreover, the nPM-driven increase of sAPPβ/α implies deficits of the neurotrophic sAPPα, which can rescue deficits of synaptic plasticity (LTP) in CA1 neurons of APP-knockout mice.^20, 21^

Given the growing literature linking air pollution with cognitive deficits across the life course, the selective neurotoxic effects on CA1 neurons underscores the possibility that PM exposure may differentially damage the medial temporal lobe-hippocampus memory system,^48^ a vulnerable neural network in both brain aging and neurodegenerative disease.^49^ Neurotoxicological and neuroimaging studies also show white matter vulnerability to PM neurotoxicity.^12, 50, 51^ The WHIMS-MRI subcohort shows associations between PM_2.5_ and smaller volumes of normal-appearing white matter in frontal and temporal lobes.^12, 52^ Total hippocampal volume did not differ by PM_2.5,_ but CA1 hippocampal subfields were not resolved. From a systems neurotoxicity perspective, these findings suggest the hypothesis that airborne PM-induced pathological brain aging may be initiated by white matter neurodegeneration, with ensuing neuroanatomical progression of AD from the entorhinal cortex in the medial temporal lobe to the hippocampus via the myelinated perforant path.^53, 54^

Our epidemiologic and experimental findings suggest that APOE ɛ4 may increase susceptibility to the adverse effects of particulate air pollutants. We followed an expert-proposed framework combining epidemiologic and toxicological evidence to make causal inference,^55^ focusing on the comparison of PM effect sizes, rather than the interaction *P*-value, which can be misleading.^56^ The adverse effects of high ambient PM_2.5_ on global cognitive decline and dementia risk were several fold greater in APOE ɛ4/4 than ɛ3/3 carriers. Because APOE4 frequencies vary widely by populations,^57^ their potential interactions with spatially varying ambient PM exposure to accelerate pathological brain aging may explain the geographic disparities in dementia incidence.^58^ Possible interactions of air pollution exposure and APOE ɛ4 on accelerated brain aging are also suggested by postmortem findings from a study in Mexico,^59^ which lacked comparison with APOE ɛ4 carriers residing in cleaner air. Neurocognitive effects of airborne particles interacting with APOE alleles were also reported for a cross-sectional study in older German women for their joint effect on constructional praxis.^60^ Interpretation is difficult because overall there were no associations across all tested domains (including episodic memory and executive function) and no adjustment for multiple comparisons. A recent case–control study in Taiwan reported an increased risk for AD associated with high ambient PM_10_, with the observed association not varying by APOE genes.^36^ This study was limited for its retrospective design prone to selection biases (for example, possible oversampling of ɛ4 carriers in controls).

We recognize several limitations of our study. First, this study of older women may not be generalized to men. Second, our study examined the association with PM_2.5_ mass, but had no information on particle constituents, emission sources, or interactions with other pollutants. Although research on cardiopulmonary end points is beginning to include these complexities of PM exposures, such data are both costly and limited for nationwide cohorts. Third, the employed spatiotemporal models only allowed estimates of late-life exposure to PM_2.5_ after 1999. As air pollution levels have been declining over the past 20 years, long-term exposure, especially during mid- or earlier life, may impart a greater risk. Finally, male mice warrant study for nPM effects on amyloid processing.

In summary, we provide clear evidence that the hazards of particulate air pollutants for brain health extend to dementia risk in a US-wide sample of older women and give, we believe, the first evidence from AD transgenic mice that exposure to urban airborne particulates can intensify amyloid accumulation and neurodegeneration. Moreover, these joint data from humans and mice provide the first evidence that neurodegenerative effects of airborne PM may involve gene-environment interactions with APOE ɛ4, the major genetic risk factor for pathological brain aging and AD. The association between PM_2.5_ exposure and increased dementia risk suggests that the global burden of disease attributable to PM_2.5_ pollution has been underestimated, especially in regions with large populations exposed to high ambient PM_2.5_.
