# Supplementary figures and images for: Particulate air pollutants, APOE alleles and their contributions to cognitive impairment in older women and to amyloidogenesis in experimental models
Source: Transl Psychiatry. 2017 Jan 31;7(1):e1022–. doi: 10.1038/tp.2016.280 (PMC5299391; doi:10.1038/tp.2016.280)

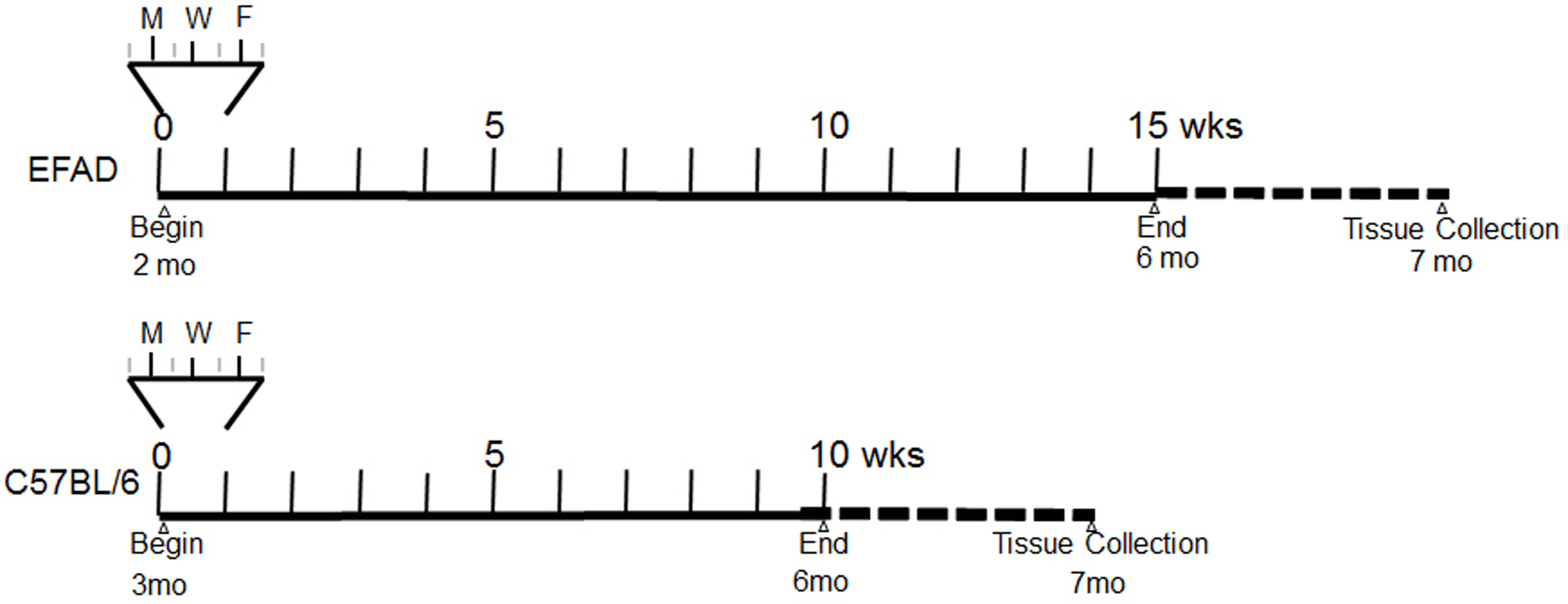

Supplement: Supplementary Figure 1 [file tp2016280x1.tif]

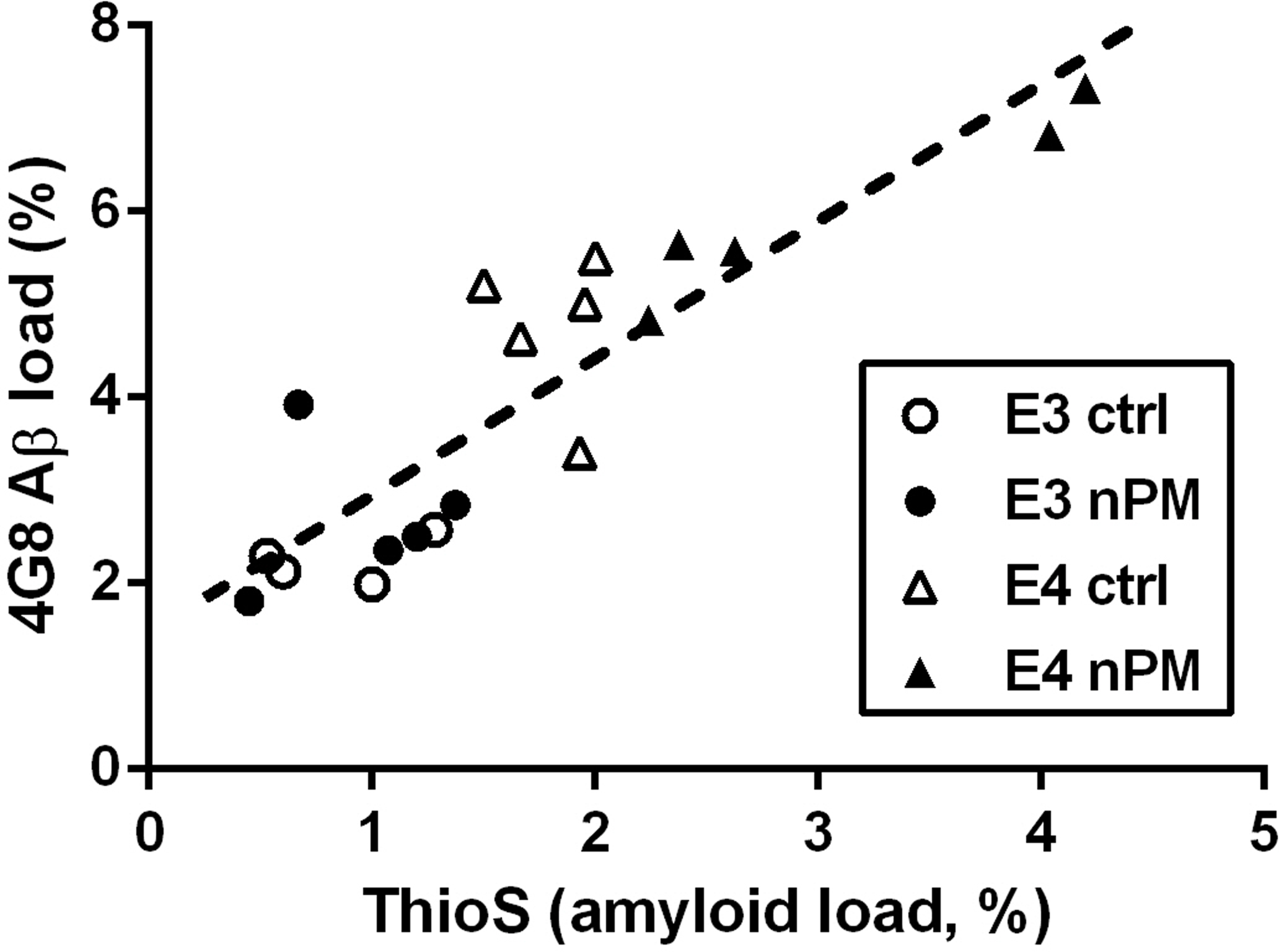

Supplement: Supplementary Figure 2 [file tp2016280x2.tif]

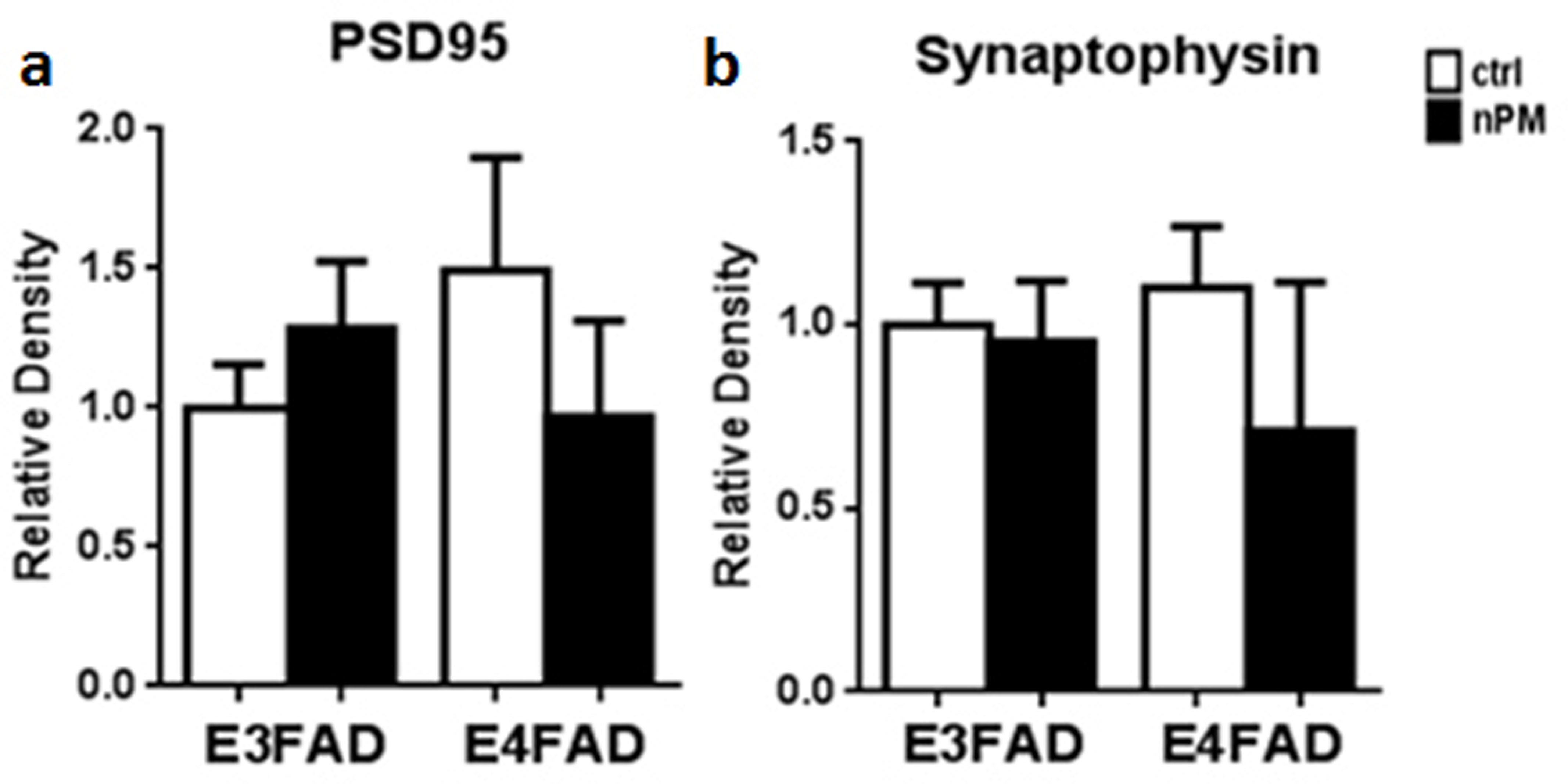

Supplement: Supplementary Figure 3 [file tp2016280x3.tif]
