# Supplementary material for: Particulate air pollutants, APOE alleles and their contributions to cognitive impairment in older women and to amyloidogenesis in experimental models
Source: Transl Psychiatry. 2017 Jan 31;7(1):e1022–. doi: 10.1038/tp.2016.280 (PMC5299391; doi:10.1038/tp.2016.280)
Supplement: Supplementary Methods [file tp2016280x4.docx]

**Supplementary Materials**

***Part A: Air-Pollution Neuroepidemiologic Study***

**Study participants**

We conducted an air pollution-neuroepidemiologic study, an observational study built on the prospective cohort of older women from the WHIMS. The WHIMS was a clinical trial designed to test the hypothesis that hormonal therapy (HT) reduces the incidence of all-cause dementia in women aged 65 and older. Community-dwelling WHIMS participants were recruited between May 28, 1995 and December 13, 1999. Eligibility criteria included participants who were aged 65 to 79 years and free of dementia defined by WHIMS protocols described elsewhere^1^.

The WHI-HT included two randomized, double-blind, placebo-controlled clinical trials of conjugated equine estrogen treatment (E-alone) for women with a prior hysterectomy or conjugated equine estrogen in combination with medroxyprogesterone acetate (E+P) for women without a hysterectomy. In July, 2002, after discovering an unfavorable risk-to-benefit ratio of its non-cognitive endpoints, the E+P trial was discontinued. The E-alone trial also ended earlier than planned in February, 2004, due to a greater risk of stroke and a lack of benefit for coronary heart disease. Although the WHIMS was an ancillary study to the WHI-HT, after the cessation of the HT trials, the WHIMS participants continued to be followed for annual cognitive assessments. In 2008, WHIMS transitioned to the WHIMS Epidemiology of Cognitive Health Outcomes (ECHO) study with annual follow-up.

**Ascertainment and selection of clinical outcomes**

The present study included two prospectively-defined outcomes: the primary endpoint was becoming an incident case of all-cause dementia and the secondary endpoint was having an accelerated decline in global cognitive function before reaching dementia. We were not able to conduct analyses restricted to AD, because of limited statistical power with a small number of events within the APOE-defined subgroups. . Our secondary outcome was the accelerated decline in global cognitive function, operationally defined as having an 8-point (~ 2 standard errors) loss in the Modified Mini-Mental State (3MS) ^2^ Examinations in two consecutive assessments. This cut-off was selected, because a decrease of 3MS by >5 points was considered a clinically significant decline in global cognitive functions^3^. These outcomes were based on standardized ascertainment, including annual screening of global cognitive function, neuropsychological and functional assessment, and collection of clinical data to rule out possible reversible causes of cognitive impairment, all concluded with central adjudication for final classification of dementia (vs. non-demented). For annual screening conducted in 49 WHIMS sites (including satellite clinics), centrally-trained/-certified and masked interviewers administered the 3MS during each clinic visit. Beginning with WHIMS ECHO in 2008, annual screening was conducted by telephone using the Telephone Interview for Cognitive Status-modified (TICSm) ^4^, which was highly correlated (0.89) with 3MS in previous studies. Women who screened positive according to age-/education-adjusted 3MS cut-points proceeded to extensive neuropsychological testing (including the Consortium to Establish a Registry for AD battery) and behavioral symptoms/function assessment. These participants subsequently received a detailed neurological examination and neuropsychiatric evaluation by board-certified physicians with experience in diagnosing dementia. Each suspected case of dementia underwent cranial CAT scan and a series of laboratory tests to rule out reversible causes of cognitive decline and dementia. For women screened positive (i.e., TICSm<31) during WHIMS ECHO follow-up, a reliable and pre-identified informant was interviewed by telephone using the standardized Dementia Questionnaire ^5^ to assess the histories of cognitive/behavioral changes, functional impairments, and health events that could have affected cognitive functioning (e.g., stroke). Data from all longitudinal assessments in the WHIMS/WHIMS ECHO and relevant supplemental information from the WHI (e.g., cardiovascular events) were then submitted to the central adjudication committee (3 board-certified neurologists/ geriatric psychiatrists with extensive experience in diagnosing dementias) for final outcome classification, based on the *Diagnostic and Statistical Manual of Mental Disorders, Fourth Edition,* which had been found to have substantial inter-rater reliability and good validity for AD (compared with post-mortem confirmation) ^6^.

**APOE genotype data**

Genetic data included in our analyses came from a WHI core study (W63) resource derived from the genome-wide association study sample, which included WHI participants with European ancestry (primarily non-Hispanic whites).  APOE genotypes were assigned, based on rs429358 and rs7412 genotype results from imputation and harmonization.  The imputation was conducted using the 1000 Genomes Project reference panel and the MaCH algorithm as implemented in Minimac (R^2^ = 0.98 for each SNP in the study population) ^7^.

**Estimation of residential exposure to fine particles**

WHIMS participants’ residential addresses, prospectively collected at each clinic visit and updated at least biannually, were geocoded following a standardized protocol ^8^. The Bayesian Maximum Entropy (BME)-based spatiotemporal modeling approach ^9^ was employed to estimate the ambient concentration of PM_2.5_ at residential locations in 1999-2010. To characterize spatiotemporal interdependence of the environmental data to estimate mean trends and covariance of the air pollution fields over space and time, the BME method integrates the nationwide monitoring data from the U.S. EPA Air Quality System (AQS) and the output of chemical transport models (CTM), entitled Community Multiscale Air Quality ([www.cmaq-model.org](http://www.cmaq-model.org)).

BME is a mathematically rigorous geostatistical space/time framework developed by Christakos.^9, 10^ The posterior probability density function $f_{K}$, which describes the process $x_{k}$ at an estimation point of interest $\boldsymbol{p}_{k}$**,** is given by the BME equation:

$f_{K}\left( x_{k} \right)= A^{-1}\int d\mathbf{x}f_{S}\left( \mathbf{x}_{\mathbf{s}} \right)f_{G}\left( \mathbf{x} \right)$,

where $\boldsymbol{x}=(x_{k},\boldsymbol{x}_{s})$ is a realization of $\boldsymbol{X}$ at points $\boldsymbol{p}=(\boldsymbol{p}_{k},\boldsymbol{p}_{s})$, $A$ is a normalization constant, $f_{G}$ is the general knowledge base, and $f_{s}$ is the site-specific knowledge base.

We define the transformation of the PM_2.5_ observed data $\boldsymbol{z}_{h}$ at locations $\boldsymbol{p}_{h}$ as $\boldsymbol{x}_{h}=\boldsymbol{z}_{h}-o_{Z}(\boldsymbol{p}_{h})$ where $o_{Z}(\boldsymbol{p})$ may be any deterministic offset that can be mathematically calculated without error as a function of the space/time coordinate $\boldsymbol{p}$. We then define $X\left( \boldsymbol{p} \right)$ as a homogeneous/stationary S/TRF representing the variability and uncertainty associated with the transformed data $\boldsymbol{x}_{h}$, and we let $Z\left( \boldsymbol{p} \right)=X\left( \boldsymbol{p} \right)+o_{Z}\left( \boldsymbol{p} \right)$ be the S/TRF representing PM_2.5_. We calculated $\hat{z}_{k}$, the estimated daily PM_2.5_ at unmonitored location $\boldsymbol{p}_{k}$ by obtaining the BME estimate $\hat{x}_{k}$ for the transformed S/TRF $X\left( \boldsymbol{p} \right)$ at the estimation point $\boldsymbol{p}_{k}$, and adding back $o_{z}(\boldsymbol{p}_{k})$, the offset calculated at $\boldsymbol{p}_{k}$.

The BME general knowledge base comes in the form of an offset and covariance model. We calculated the offset using a space/time composite kernel smoothing of the data and calculated the covariance using a two-structured exponential model in space and time. The BME site-specific knowledge comes from observed data and CTM-modeled PM_2.5_ data. The CTM data have quantifiable uncertainty with through the probability density function $f(z_{i}|\tilde{z}_{i},\boldsymbol{p}_{i})$ where $f(z_{i}|\tilde{z}_{i},\boldsymbol{p}_{i})$ is Gaussian distributed with mean $\lambda_{1}\left( \tilde{z}_{i},\boldsymbol{p}_{i} \right)$ and variance $\lambda_{2}\left( \tilde{z}_{i},\boldsymbol{p}_{i} \right)$, with $\tilde{z}_{i}$ being the CTM data at $\boldsymbol{p}_{i}$. The parameters $\lambda_{1}\left( \tilde{z}_{i},\boldsymbol{p}_{i} \right)$ and $\lambda_{2}\left( \tilde{z}_{i},\boldsymbol{p}_{i} \right)$ are dependent on the modeled value within a given grid and are estimated by extending a space/time component from previous work. The resulting CTM data are then error-corrected. In previous work error-correction was only a function of $\tilde{z}_{i}$ and was fixed across its domain both spatially and temporally. This work allows error-correction to not only change with $\tilde{z}_{i}$, but also as a function on space and time.

To assess the estimation accuracy of our BME model, a 10-fold estimation analysis was performed, with the AQS monitoring stations evenly divided in 10 distinct sets. For each fold, the BME estimation was implemented to obtain the daily estimates using only the data from the remaining 90% of the monitoring stations. Our empirical data showed that BME estimates of daily PM_2.5_ exposures correlated (with average Pearson’s $r^{2}=0.70$) well with the AQS recorded concentrations. This statistically-validated BME model was applied to each geocoded residential location to generate a yearly time-series of PM_2.5_ exposure, and then combined with the residential histories including relocations to calculate the 3-year moving average PM_2.5_ exposures. The exposure period was classified as “high” if the 3-year average PM_2.5_ exceeded (>12 µg/m^3^) the current National Ambient Air Quality Standards ^11^. The AQS has not started monitoring nPM. The constructed BME models allowed us to estimate late-life exposure to PM_2.5_ in 1999-2010, but there were no appropriate PM_2.5_ monitoring data and chemical transport models before 1999 to estimate the exposures likely with higher ambient levels when WHIMS participants were aged less than 65 years. Also, monitoring data on PM_2.5_ constituents or emission sources did not have the adequate quality and sufficient geographic coverage needed to estimate the nationwide residential location-specific ambient levels in 1999-2010.

**Relevant covariates**

At WHI baseline, WHIMS participants completed structured questionnaires regarding demographics (age, race-ethnicity), socioeconomic status (SES, including education in years, family income, employment status), lifestyle factors (smoking, alcohol consumption, physical activity), and relevant clinical characteristics (prior use of menopausal HT, depression, cardiovascular disease and related risk factors). The Burnam screening algorithm ^12^ was used to characterize the likelihood of prior depressive disorders. Women were grouped according to body mass index (in kg/m^2^) categories (< 25.0 vs. 25.0-29.9 vs. ≥30.0). Hypertension was defined as antihypertensive medication or elevated blood pressure (systolic≥140 or diastolic≥90 mmHg). Treated diabetes mellitus was defined as a physician diagnosis plus oral medications or insulin therapy. History of cardiovascular disease included previous coronary heart disease (myocardial infarction, coronary angioplasty, or coronary artery bypass graft), stroke, or transient ischemic attack. Good reliability and validity of both the physical measures and self-reported medical histories have been documented ^13^.

**Statistical analysis**

Of 4504 WHIMS participants assigned with APOE genotypes, 857 were non-eligible (717 with ԑ2/2, ԑ2/3 or ԑ2/4 plus 140 with PM_2.5_ data missing) and thus excluded in the present study. For the remaining 3647 older women, we compared the distributions of estimated PM_2.5_ exposures across different population characteristics, using chi-square tests. The PM_2.5_ exposures distributions were based on the individual-specific summary measure aggregated during the study follow-up in 1999-2010. We examined the distribution of 3-year PM_2.5_ exposures in relation to other selected personal and clinical characteristics (Table S1). Cox proportional hazard models were used to estimate hazard ratios (HRs) and 95% confidence intervals (CIs) for adverse events associated with estimated time-varying 3-year average PM_2.5_ exposures, adjusting for potential confounders, including age, geographic region, education, income, employment status, lifestyle factors (smoking; alcohol use; physical activities), and clinical characteristics (use of hormone treatment; depression; body mass index; hypercholesterolemia; hypertension, diabetes; and histories of cardiovascular disease). We used the directed acyclic graph to identify a list of important covariates *a priori* as potential confounders for the putative neurotoxic effects of PM exposures. Conceptually, these covariates would have established causal relationships *both* with the studied outcomes (global cognitive decline; risk of dementia), *and* also with the PM exposures. Because we relied on residential locations to define the primary exposure variables, population characteristics or personal attributes that determine where the people live (and thus the estimated exposure levels) in late life could be considered as potential confounders if they are also known to affect the studied outcomes. For epidemiologic studies on brain health and outdoor air pollutants that relied on location-based exposure estimation, rigorous accounting for such potential confounding had been advocated ^14^. In this regard, age, race/ethnicity, socioeconomic status, and lifestyle factors (including smoking, alcohol consumption, and physical activities) should be conceptualized as potential confounders *a priori* and included in the fully adjusted analyses. Also, because there is increasing evidence of geographic differences in dementia incidences ^15^, to control for possible spatial confounding, possible geographic differences were accounted for in the Cox regression models, jointly with fixed effects (to denote the regional difference across US census-defined geographic region) and spatial random effects ^16^ (to represent the differences by study sites).

| **Table S1: Population Characteristics in Relation to Long-Term Exposure to Fine Particulate Matter (1999-2010) in WHIMS Subcohort** | | | | | | | | |
| --- | --- | --- | --- | --- | --- | --- | --- | --- |
| **Population Characteristics** |  | | **3-Year**  **Average PM_2.5_** | **Quartile of 3-Year PM_2.5_ (in µg/m^3^)** | | | |  |
|  |  | | **mean ± SD**  **(in µg/m^3^)** | **3.97-10.62 (median=9.87)** | **10.62-12.19 (median=11.26)** | **12.19-14.34 (median=13.31)** | **14.34-22.55 (median=15.72)** | **p-value** |
| **All** | **N=3647** | | **12.50 ± 2.69** | **N=912** | **N=912** | **N=912** | **N=911** |  |
| **U. S. Region** |  | |  |  |  |  |  | <.0001 |
| Northeast | 1037 | | 12.1 ± 1.67 | 210 (20.3%) | 366 (35.3%) | 369 (35.6%) | 92 (8.9%) |  |
| South | 715 | | 13.45 ± 2.09 | 112 (15.7%) | 83 (11.6%) | 269 (37.6%) | 251 (35.1%) |  |
| Midwest | 898 | | 12.95 ± 2.27 | 179 (19.9%) | 207 (23.1%) | 210 (23.4%) | 302 (33.6%) |  |
| West | 997 | | 11.84 ± 3.8 | 411 (41.2%) | 256 (25.7%) | 64 (6.4%) | 266 (26.7%) |  |
| **Age group at screening** |  | |  |  |  |  |  | 0.001 |
| 63-69 | 1713 | | 12.46 ± 2.65 | 436 (25.5%) | 420 (24.5%) | 445 (26%) | 412 (24.1%) |  |
| 70-74 | 1353 | | 12.41 ± 2.7 | 354 (26.2%) | 342 (25.3%) | 343 (25.4%) | 314 (23.2%) |  |
| ≥75 | 581 | | 12.84 ± 2.79 | 122 (21%) | 150 (25.8%) | 124 (21.3%) | 185 (31.8%) |  |
| **Family Income** |  | |  |  |  |  |  | 0.03 |
| Less than $10,000 | 144 | | 12.27 ± 2.65 | 35 (24.3%) | 47 (32.6%) | 26 (18.1%) | 36 (25%) |  |
| $10,000 to $34,999 | 1734 | | 12.42 ± 2.73 | 469 (27%) | 420 (24.2%) | 408 (23.5%) | 437 (25.2%) |  |
| $35,000 to $74,999 | 1251 | | 12.56 ± 2.68 | 301 (24.1%) | 318 (25.4%) | 327 (26.1%) | 305 (24.4%) |  |
| ≥$75,000 | 383 | | 12.8 ± 2.64 | 78 (20.4%) | 89 (23.2%) | 112 (29.2%) | 104 (27.2%) |  |
| Missing | 135 | | 12.48 ± 2.59 | 29 (21.5%) | 38 (28.1%) | 39 (28.9%) | 29 (21.5%) |  |
| **Education** |  | |  |  |  |  |  | 0.23 |
| <High School | 187 | | 12.62 ± 2.72 | 48 (25.7%) | 42 (22.5%) | 41 (21.9%) | 56 (29.9%) |  |
| High school/GED | 794 | | 12.47 ± 2.59 | 198 (24.9%) | 214 (27%) | 178 (22.4%) | 204 (25.7%) |  |
| >High school | 2657 | | 12.5 ± 2.72 | 666 (25.1%) | 654 (24.6%) | 690 (26%) | 647 (24.4%) |  |
| **Employment** |  | |  |  |  |  |  | 0.22 |
| Currently employed | 661 | | 12.63 ± 2.69 | 162 (24.5%) | 153 (23.1%) | 182 (27.5%) | 164 (24.8%) |  |
| Not working | 355 | | 12.64 ± 2.79 | 83 (23.4%) | 78 (22%) | 100 (28.2%) | 94 (26.5%) |  |
| Retired | 2619 | | 12.45 ± 2.68 | 667 (25.5%) | 676 (25.8%) | 626 (23.9%) | 650 (24.8%) |  |
| **Moderate/Strenuous activities ≥20 minutes** | | | |  |  |  |  | 0.05 |
| No activity | | 2051 | 12.57 ± 2.71 | 497 (24.2%) | 505 (24.6%) | 502 (24.5%) | 547 (26.7%) |  |
| Some activity | | 168 | 12.75 ± 2.45 | 30 (17.9%) | 52 (31%) | 43 (25.6%) | 43 (25.6%) |  |
| 2-4 episodes/week | | 741 | 12.38 ± 2.7 | 195 (26.3%) | 195 (26.3%) | 186 (25.1%) | 165 (22.3%) |  |
| >4 episodes/week | | 679 | 12.38 ± 2.68 | 188 (27.7%) | 158 (23.3%) | 178 (26.2%) | 155 (22.8%) |  |
| **Smoking status** | |  |  |  |  |  |  | 0.42 |
| Never Smoked | | 1912 | 12.55 ± 2.7 | 469 (24.5%) | 484 (25.3%) | 457 (23.9%) | 502 (26.3%) |  |
| Past Smoker | | 1467 | 12.44 ± 2.71 | 377 (25.7%) | 361 (24.6%) | 388 (26.4%) | 341 (23.2%) |  |
| Current Smoker | | 219 | 12.51 ± 2.65 | 56 (25.6%) | 54 (24.7%) | 52 (23.7%) | 57 (26%) |  |
| **Alcohol intake** | |  |  |  |  |  |  | 0.0002 |
| Non drinker | | 415 | 12.93 ± 2.67 | 93 (22.4%) | 79 (19%) | 107 (25.8%) | 136 (32.8%) |  |
| Past drinker | | 599 | 12.63 ± 2.83 | 154 (25.7%) | 141 (23.5%) | 132 (22%) | 172 (28.7%) |  |
| <1 drink per day | | 2098 | 12.4 ± 2.66 | 531 (25.3%) | 564 (26.9%) | 526 (25.1%) | 477 (22.7%) |  |
| >1 drink per day | | 509 | 12.43 ± 2.65 | 129 (25.3%) | 122 (24%) | 139 (27.3%) | 119 (23.4%) |  |
| **HT use ever** | |  |  |  |  |  |  | <.0001 |
| No | | 2003 | 12.57 ± 2.51 | 449 (22.4%) | 523 (26.1%) | 553 (27.6%) | 478 (23.9%) |  |
| Yes | | 1643 | 12.43 ± 2.9 | 462 (28.1%) | 389 (23.7%) | 359 (21.9%) | 433 (26.4%) |  |
| Body mass Index (kg/m^2^) | |  |  |  |  |  |  | 0.87 |
| <25 | | 1119 | 12.53 ± 2.72 | 284 (25.4%) | 267 (23.9%) | 293 (26.2%) | 275 (24.6%) |  |
| 25-29 | | 1341 | 12.55 ± 2.72 | 330 (24.6%) | 347 (25.9%) | 329 (24.5%) | 335 (25%) |  |
| ≥30 | | 1169 | 12.42 ± 2.64 | 298 (25.5%) | 293 (25.1%) | 282 (24.1%) | 296 (25.3%) |  |
| **History of depression** | |  |  |  |  |  |  | 0.40 |
| No | | 3313 | 12.5 ± 2.68 | 824 (24.9%) | 846 (25.5%) | 819 (24.7%) | 824 (24.9%) |  |
| Yes | | 268 | 12.59 ± 2.77 | 64 (23.9%) | 59 (22%) | 77 (28.7%) | 68 (25.4%) |  |
| **Diabetes treated ever (pills or shots)** | | |  |  |  |  |  | 0.14 |
| No | | 3442 | 12.52 ± 2.71 | 866 (25.2%) | 848 (24.6%) | 863 (25.1%) | 865 (25.1%) |  |
| Yes | | 201 | 12.23 ± 2.44 | 45 (22.4%) | 64 (31.8%) | 48 (23.9%) | 44 (21.9%) |  |
| **High cholesterol requiring pills ever** | | |  |  |  |  |  | 0.55 |
| No | | 2910 | 12.47 ± 2.7 | 737 (25.3%) | 733 (25.2%) | 723 (24.8%) | 717 (24.6%) |  |
| Yes | | 698 | 12.63 ± 2.67 | 164 (23.5%) | 168 (24.1%) | 181 (25.9%) | 185 (26.5%) |  |
| **Hypertension ever** | |  |  |  |  |  |  | 0.38 |
| No | | 2307 | 12.5 ± 2.69 | 591 (25.6%) | 557 (24.1%) | 580 (25.1%) | 579 (25.1%) |  |
| Yes | | 1308 | 12.5 ± 2.69 | 313 (23.9%) | 348 (26.6%) | 322 (24.6%) | 325 (24.8%) |  |
| **Cardiovascular disease** | |  |  |  |  |  |  | 0.48 |
| No | | 3028 | 12.49 ± 2.7 | 767 (25.3%) | 769 (25.4%) | 751 (24.8%) | 741 (24.5%) |  |
| Yes | | 578 | 12.5 ± 2.65 | 141 (24.4%) | 133 (23%) | 150 (26%) | 154 (26.6%) |  |
| **Subcohort membership: E-alone** | |  |  |  |  |  |  | 0.28 |
| E-alone control | | 609 | 12.35 ± 2.74 | 159 (26.1%) | 161 (26.4%) | 136 (22.3%) | 153 (25.1%) |  |
| E-alone intervention | | 588 | 12.57 ± 2.77 | 156 (26.5%) | 128 (21.8%) | 145 (24.7%) | 159 (27%) |  |
| **Subcohort membership: E+P** | |  |  |  |  |  |  | 0.42 |
| E+P control | | 1251 | 12.51 ± 2.7 | 301 (24.1%) | 317 (25.3%) | 339 (27.1%) | 294 (23.5%) |  |
| E+P intervention | | 1199 | 12.53 ± 2.62 | 296 (24.7%) | 306 (25.5%) | 292 (24.4%) | 305 (25.4%) |  |
| **APOE genetic category** | |  |  |  |  |  |  | 0.48 |
| e3/e3 | | 2644 | 12.51 ± 2.67 | 671 (25.4%) | 647 (24.5%) | 673 (25.5%) | 653 (24.7%) |  |
| e3/e4 | | 922 | 12.49 ± 2.76 | 222 (24.1%) | 248 (26.9%) | 214 (23.2%) | 238 (25.8%) |  |
| e4/e4 | | 81 | 12.48 ± 2.79 | 19 (23.5%) | 17 (21%) | 25 (30.9%) | 20 (24.7%) |  |
| * defined as the subject-specific average of time-varying 3-year PM_2.5_ exposures during the follow-up time prior to dementia event time or censoring time | | | | | | | | |

**Table S2: Cox Proportional Hazard Models for the Adverse Effects(†) of PM_2.5_ on Accelerated Global Cognitive Decline and Increased Risk for All-Cause Dementia, Stratified by APOE Genotypes in Non-Hispanic White Subcohort(‡) of WHIMS, 1999-2010**

|  |  | **Accelerated Global Decline** | **All-Cause Dementia** |
| --- | --- | --- | --- |
| **Models** |  | **Hazard Ratio (95% CI)** | **Hazard Ratio (95% CI)** |
| **I** | **Main PM_2.5_ Effect** |  |  |
|  | High vs. Low PM_2.5_ | 1.83 (1.47, 2.27); P<0.01 | 1.67 (1.21, 2.30); P<0.01 |
|  | **PM_2.5_ Effect by APOE** |  |  |
|  | ε3/3 | 1.71 (1.31, 2.23); P<0.01 | 1.26 (0.78, 2.03); P=0.35 |
|  | ε3/4 | 1.96 (1.35, 2.84); P<0.01 | 1.89 (1.23, 2.91) P<0.01 |
|  | ε4/4 | 2.87 (1.14, 7.23); P=0.02 | 3.53 (1.21, 10.34) P=0.02 |
|  |  | Interaction p=0.52 | Interaction p=0.16 |
| **II** | **Main PM_2.5_ Effect** |  |  |
|  | High vs. Low PM_2.5_ | 1.85 (1.45, 2.36); P<0.01 | 1.71 (1.20, 2.45); P<0.01 |
|  | **PM_2.5_ Effect by APOE** |  |  |
|  | ε3/3 | 1.71 (1.28, 2.28); P<0.01 | 1.36 (0.81, 2.28); P=0.24 |
|  | ε3/4 | 2.03 (1.36, 3.02); P<0.01 | 1.88 (1.17, 3.01); P<0.01 |
|  | ε4/4 | 2.73 (1.08, 6.94); P=0.03 | 3.19 (1.06, 9.57); P=0.04 |
|  |  | Interaction p=0.54 | Interaction p=0.31 |
| **III** | **Main PM_2.5_ Effect** |  |  |
| (Fig.1: Adjusted) | High vs. Low PM_2.5_ | 1.81 (1.42, 2.32); P<0.01 | 1.92 (1.31, 2.80); P<0.01 |
|  | **PM_2.5_ Effect by APOE** |  |  |
|  | ε3/3 | 1.65 (1.23, 2.23); P<0.01 | 1.68 (0.97, 2.92); P=0.06 |
|  | ε3/4 | 1.93 (1.29, 2.90); P<0.01 | 1.91 (1.17, 3.14); P=0.01 |
|  | ε4/4 | 3.64 (1.36, 9.69); P<0.01 | 3.95 (1.18, 13.19); P=0.03 |
|  |  | Interaction p=0.29 | Interaction p=0.43 |

† expressed as the hazard ratio (95% confidence interval) associated with increased time-varying PM_2.5_ comparing high (3-year average >12 µg/m^3^) to low (3-year average ≤12 µg/m^3^) exposure

‡subcohort of 3647 non-Hispanic white women with ε3/3, ε3/4, or ε4/4

Model I adjusting APOE genotype

Model II adjusting for APOE genotype, age, geographic region/spatial random effect, SES (participant’s education and family income), and lifestyle factors (alcohol consumption status, smoking status and physical activities)

Model III adjusting for Model-II covariates plus, body mass index, and prior depression, cardiovascular disease histories, and conventional cardiovascular disease risk factors (hypertension; diabetes mellitus; hypercholesterolemia)

**Table S3. Statistical analysis of mouse data.**

|  |  | **EFAD** | | | | | | | | **C57BL/6** | |
| --- | --- | --- | --- | --- | --- | --- | --- | --- | --- | --- | --- |
|  | **Endpoint** | **APOE4 effect** | | **nPM exposure effect, overall** | | **nPM exposure effect, by genotype** | | | |  |  |
|  |  |  |  |  |  | **E3FAD** | | **E4FAD** | |  |  |
|  |  | **Coeff** | **p-value** | **Coeff** | **p-value** | **Coeff** | **p-value** | **Coeff** | **p-value** | **Coeff** | **p-value** |
| **cortex** | **Aβ oligomers** | ***0.06*** | ***0.0001*** | ***0.05*** | ***0.0001*** | 0.07 | 0.07 | ***0.03*** | ***0.03*** | - | - |
|  | **Aβ plaque load** | ***2.88*** | ***0.001*** | ***0.97*** | ***0.02*** | 0.49 | 0.27 | ***1.39*** | ***0.04*** | - | - |
|  | **ThioS amyloid load** | ***1.44*** | ***0.001*** | ***0.53*** | ***0.12*** | -0.09 | 0.97 | ***1.06*** | ***0.001*** | - | - |
| **Hippocampus** | **GluR1** | -0.11 | 0.1 | ***-0.29*** | ***0.0001*** | ***-0.26*** | ***0.01*** | ***-0.32*** | ***0.01*** | -- | - |
|  | **GluR2** | -0.020 | 0.69 | -0.043 | 0.54 | 0.049 | 0.68 | -0.12 | 0.14 | - | - |
|  | **CA1 neurites** | -0.01 | 0.06 | ***-0.06*** | ***0.02*** | ***-0.09*** | ***0.03*** | -0.04 | 0.22 | **-0.25** | **0.003** |
|  | **DG neurites** | 0.16 | 0.94 | -0.39 | 0.85 | -1.04 | 0.75 | 0.25 | 0.91 | -0.19 | 0.23 |

CA1, region 1 of hippocampal proper; DG, dentate gyrus. Statistically significant: bold italics.

**
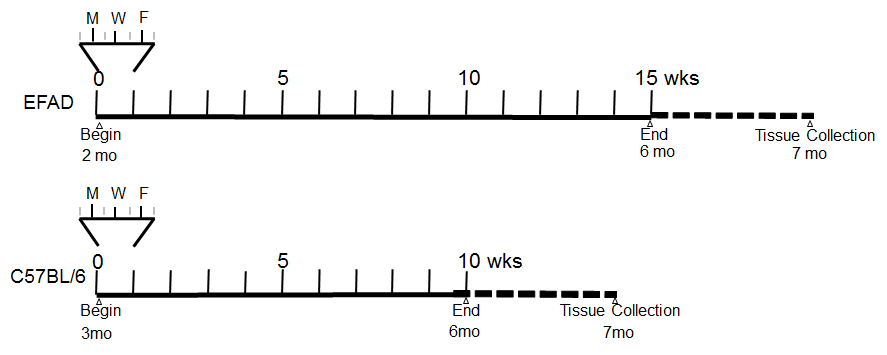
**

**Supplementary Figure 1. Timeline of mice exposure.** Alternate day intermittent exposure schedule is expanded for first week, with M (Monday) W (Wednesday) F (Friday) exposure days indicated in bold. Age of mice at beginning and end of experiment are labeled below the timeline. a. EFAD mice 15 wks exposure; b. C57BL/6 mice 10 wks exposure.

**
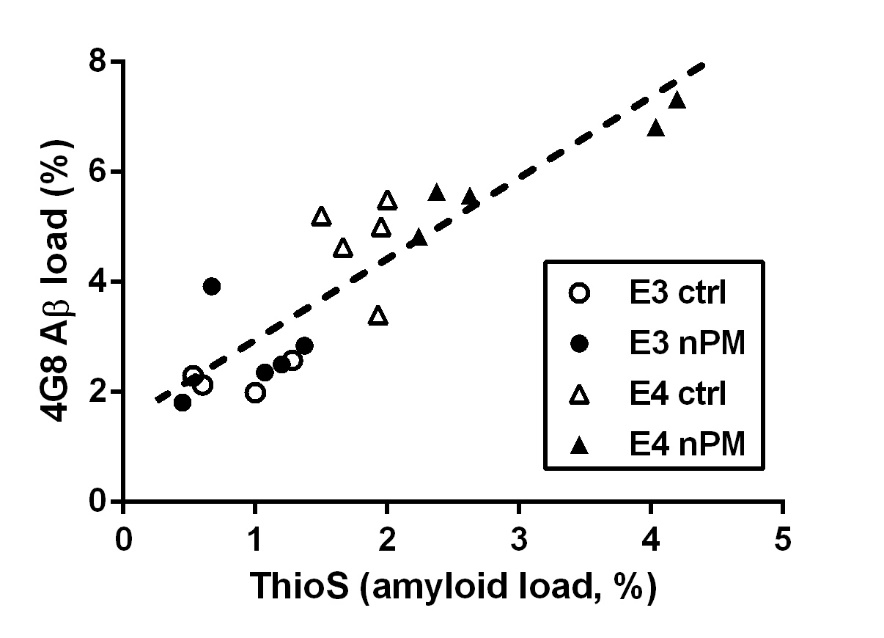
**

**Supplementary Figure 2. Correlation.** Correlation analysis of amyloid load as analyzed by Thioflavin S staining (ThioS) and Aβ plaque load as analyzed by 4G8 immunostaining is shown. r^2^=0.78, p<0.0001. open symbol: controls; black symbol: nPM exposed mice; circle: E3FAD mice; triangle: E4FAD mice.


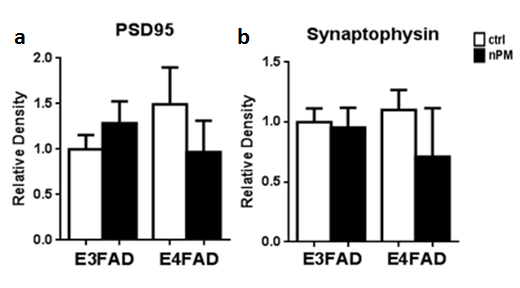


**Supplementary Figure 3. Synaptic proteins did not change after nPM exposure in female EFAD mice.** nPM exposure did not alter post-synaptic PSD95 or pre-synaptic protein synaptophysin. White bar, control; black bar, nPM exposed. N=5 mice/experimental group; Mean ± SE.

**Supplementary References**

1. Shumaker SA, Legault C, Kuller L, Rapp SR, Thal L, Lane DS *et al.* Conjugated equine estrogens and incidence of probable dementia and mild cognitive impairment in postmenopausal women: Women's Health Initiative Memory Study. *JAMA* 2004; **291**(24)**:** 2947-2958.

2. Teng EL, Chui HC. The Modified Mini-Mental State (3MS) examination. *J Clin Psychiatry* 1987; **48**(8)**:** 314-318.

3. Yaffe K, Laffan AM, Harrison SL, Redline S, Spira AP, Ensrud KE *et al.* Sleep-disordered breathing, hypoxia, and risk of mild cognitive impairment and dementia in older women. *JAMA* 2011; **306**(6)**:** 613-619.

4. Brandt J, Spencer M, Folstein M. The Telephone Interview for Cognitive Status. *Neuropsychiatr Neuropsychol Behav Neurol* 1988; **1**(2)**:** 111-117.

5. Ellis RJ, Jan K, Kawas C, Koller WC, Lyons KE, Jeste DV *et al.* Diagnostic validity of the dementia questionnaire for Alzheimer disease. *Arch Neurol* 1998; **55**(3)**:** 360-365.

6. Hogervorst E, Bandelow S, Combrinck M, Irani SR, Smith AD. The validity and reliability of 6 sets of clinical criteria to classify Alzheimer's disease and vascular dementia in cases confirmed post-mortem: added value of a decision tree approach. *Dement Geriatr Cogn Disord* 2003; **16**(3)**:** 170-180.

7. Howie B, Fuchsberger C, Stephens M, Marchini J, Abecasis GR. Fast and accurate genotype imputation in genome-wide association studies through pre-phasing. *Nat Genet* 2012; **44**(8)**:** 955-959.

8. Whitsel EA, Rose KM, Wood JL, Henley AC, Liao D, Heiss G. Accuracy and repeatability of commercial geocoding. *Am J Epidemiol* 2004; **160**(10)**:** 1023-1029.

9. Christakos G, Bogaert P, Serre ML. *Temporal GIS: advanced functions for field-based applications*. Springer: Berlin; New York, 2001, xii, 217 p. : ill. ; 224 cm. + 211 CD-ROM (214 213/214 in.)pp.

10. Christakos G. *Modern spatiotemporal geostatistics*. Oxford University Press: Oxford; New York, 2000.

11. EPA. National Ambient Air Quality Standards for Particulate Matter; Proposed Rule. In: (EPA) UEPA (ed). vol. Vol.77. Federal Register2012, pp 38890-39055.

12. Goveas JS, Espeland MA, Woods NF, Wassertheil-Smoller S, Kotchen JM. Depressive symptoms and incidence of mild cognitive impairment and probable dementia in elderly women: the Women's Health Initiative Memory Study. *J Am Geriatr Soc* 2011; **59**(1)**:** 57-66.

13. Heckbert SR, Kooperberg C, Safford MM, Psaty BM, Hsia J, McTiernan A *et al.* Comparison of self-report, hospital discharge codes, and adjudication of cardiovascular events in the Women's Health Initiative. *Am J Epidemiol* 2004; **160**(12)**:** 1152-1158.

14. Block ML, Elder A, Auten RL, Bilbo SD, Chen H, Chen JC *et al.* The outdoor air pollution and brain health workshop. *Neurotoxicology* 2012; **33**(5)**:** 972-984.

15. Russ TC, Batty GD, Hearnshaw GF, Fenton C, Starr JM. Geographical variation in dementia: systematic review with meta-analysis. *Int J Epidemiol* 2012; **41**(4)**:** 1012-1032.

16. Ripatti S, Palmgren J. Estimation of multivariate frailty models using penalized partial likelihood. *Biometrics* 2000; **56**(4)**:** 1016-1022.
